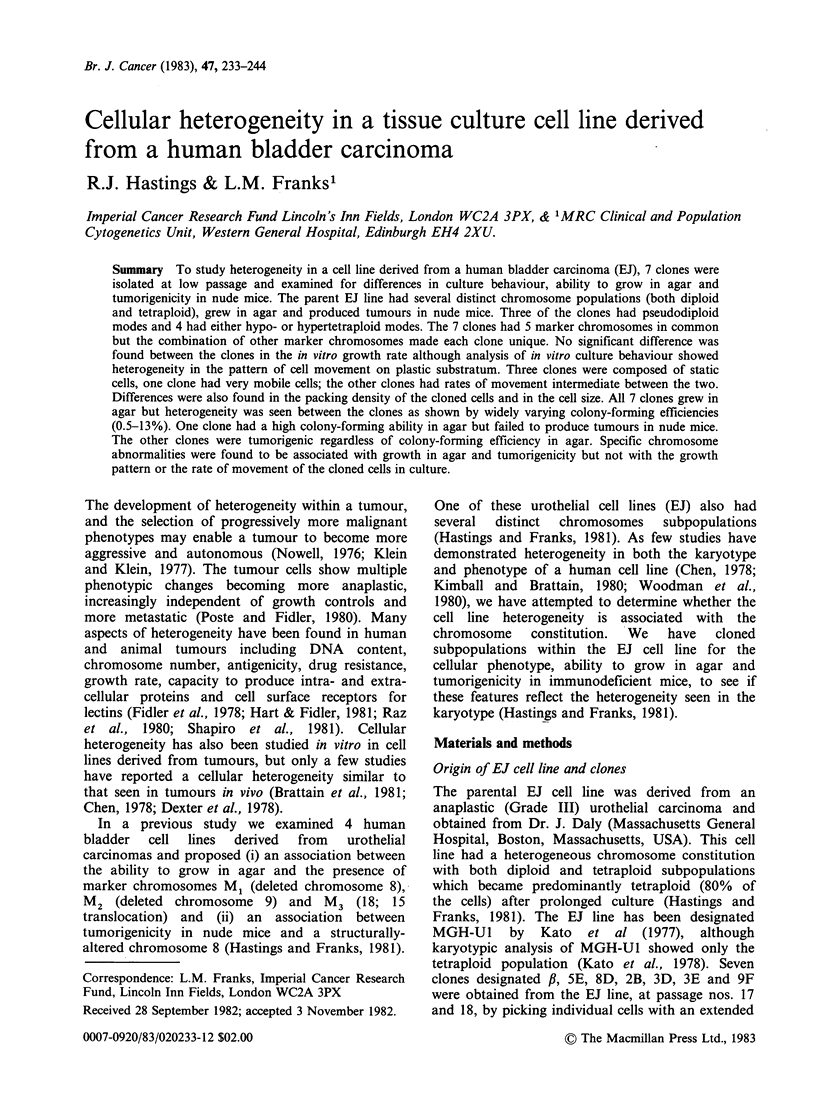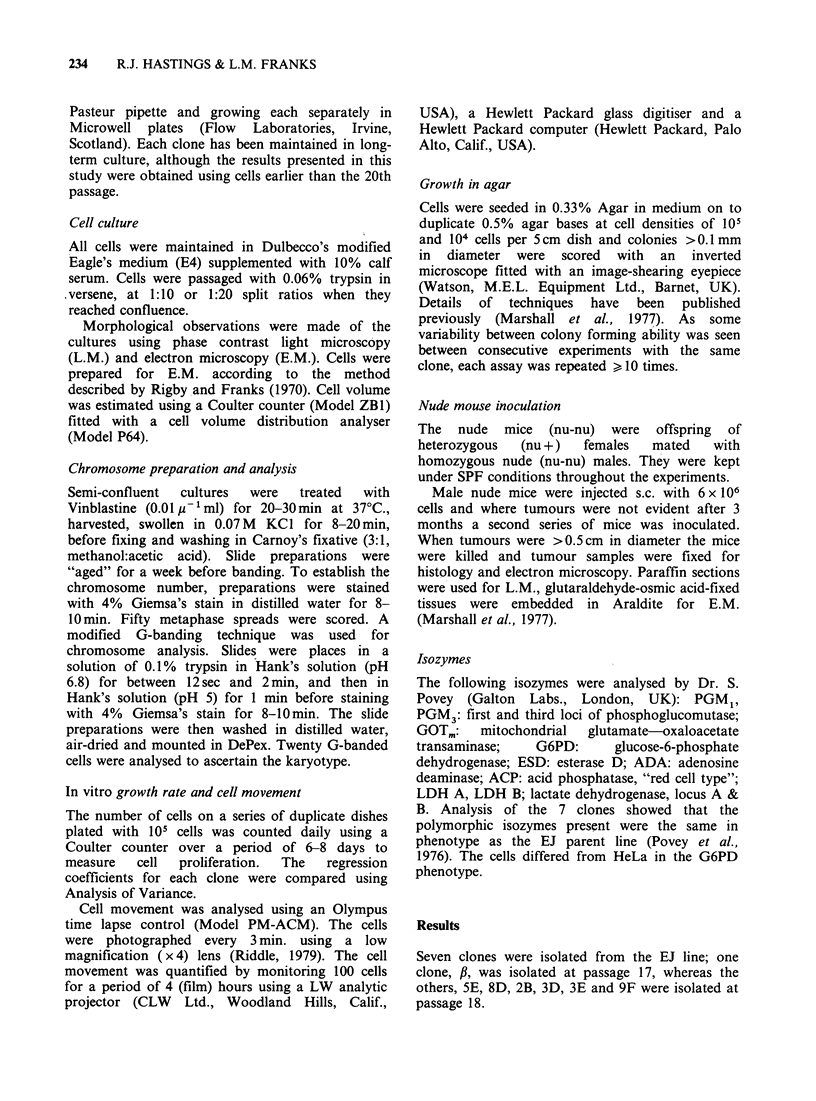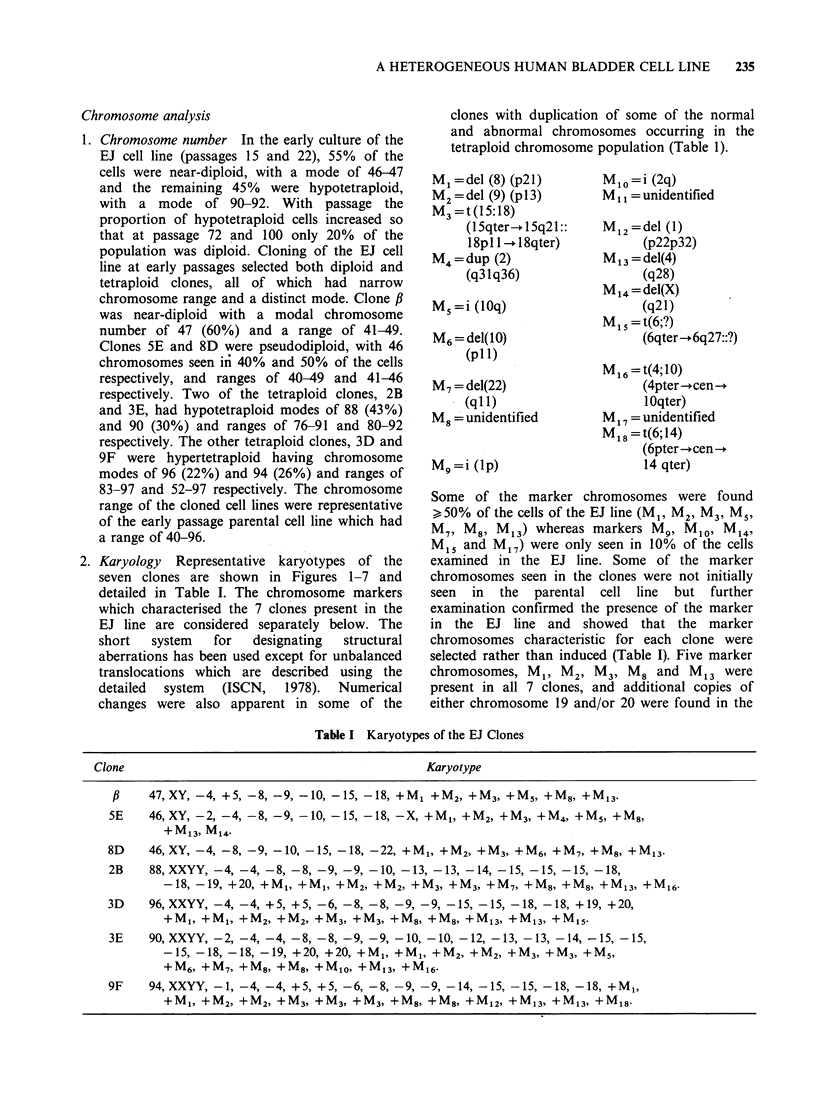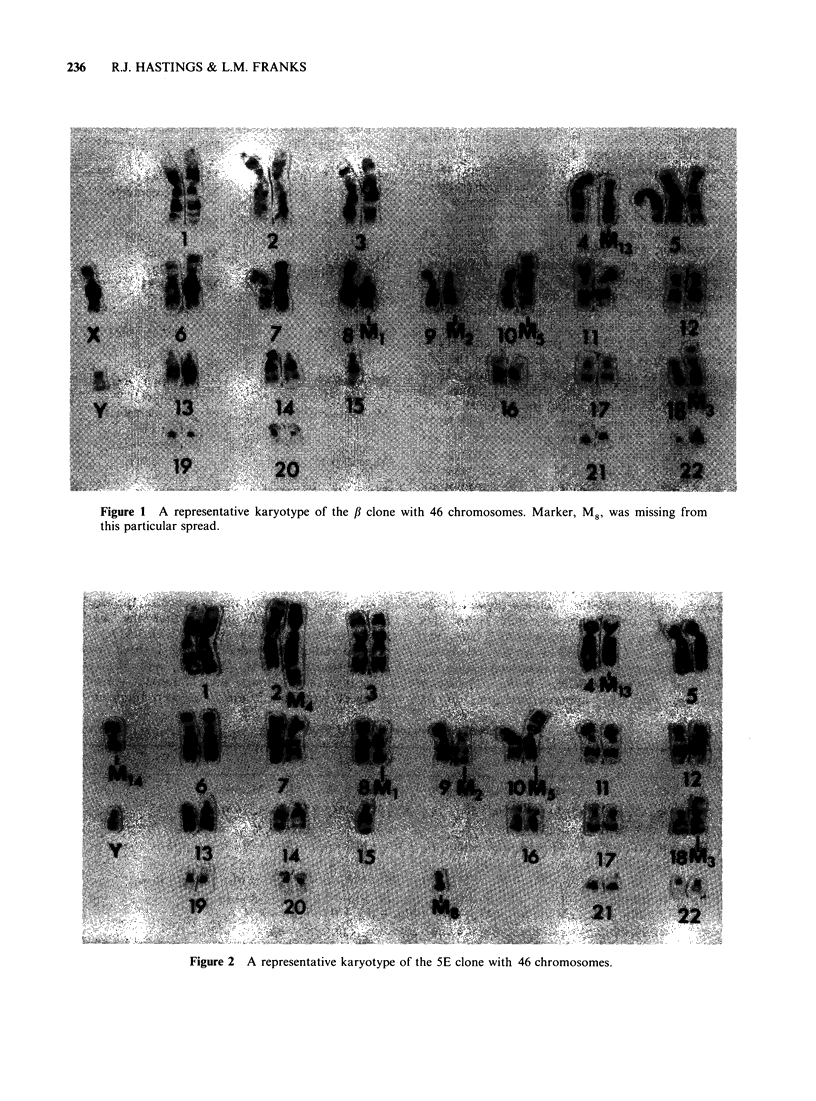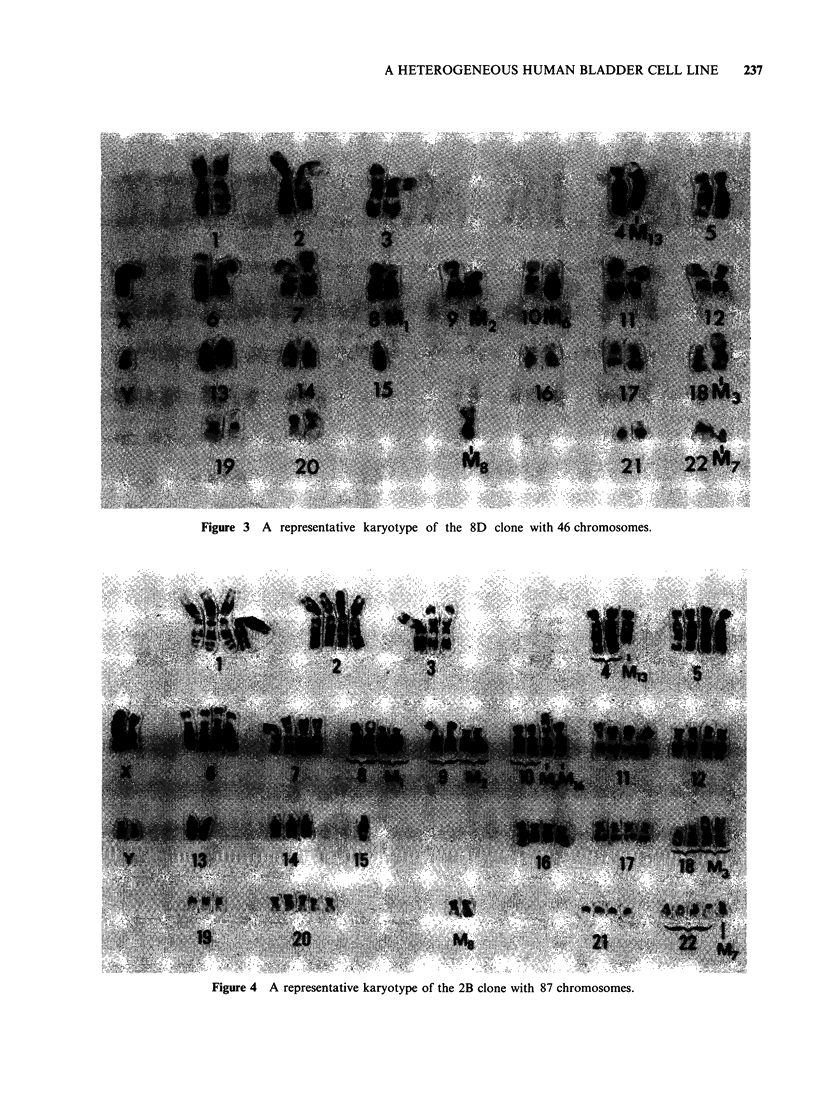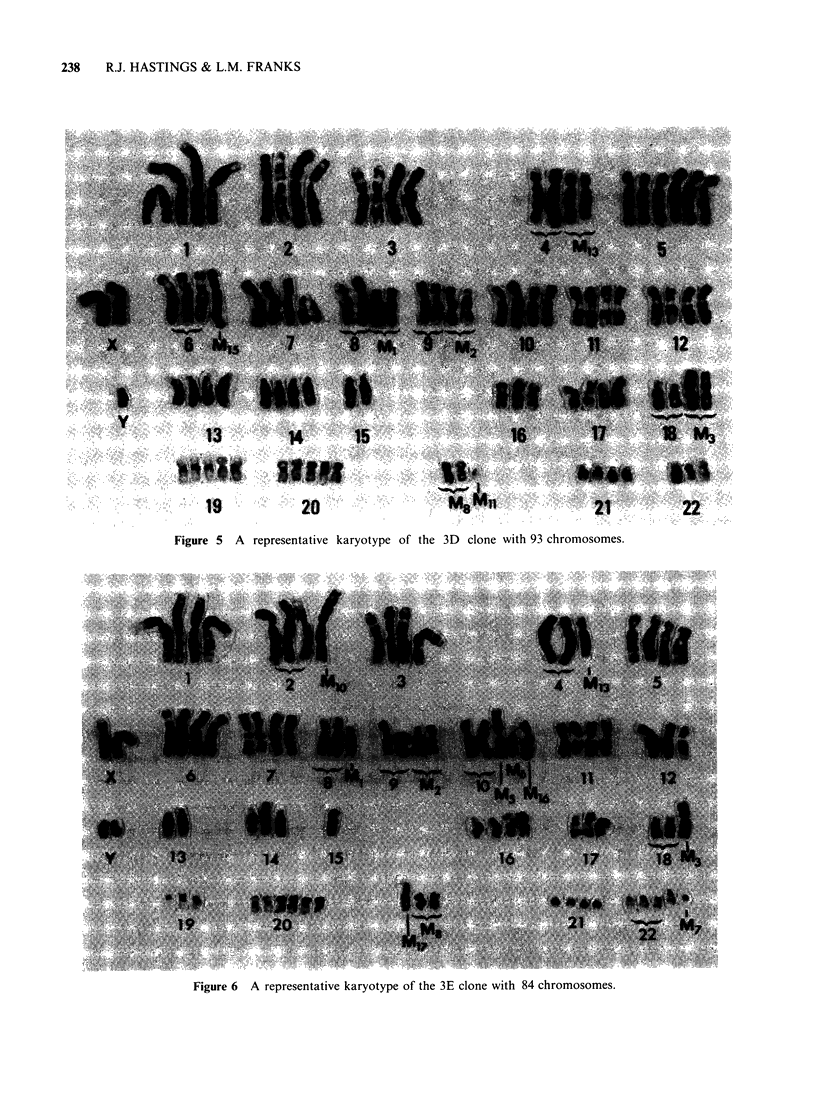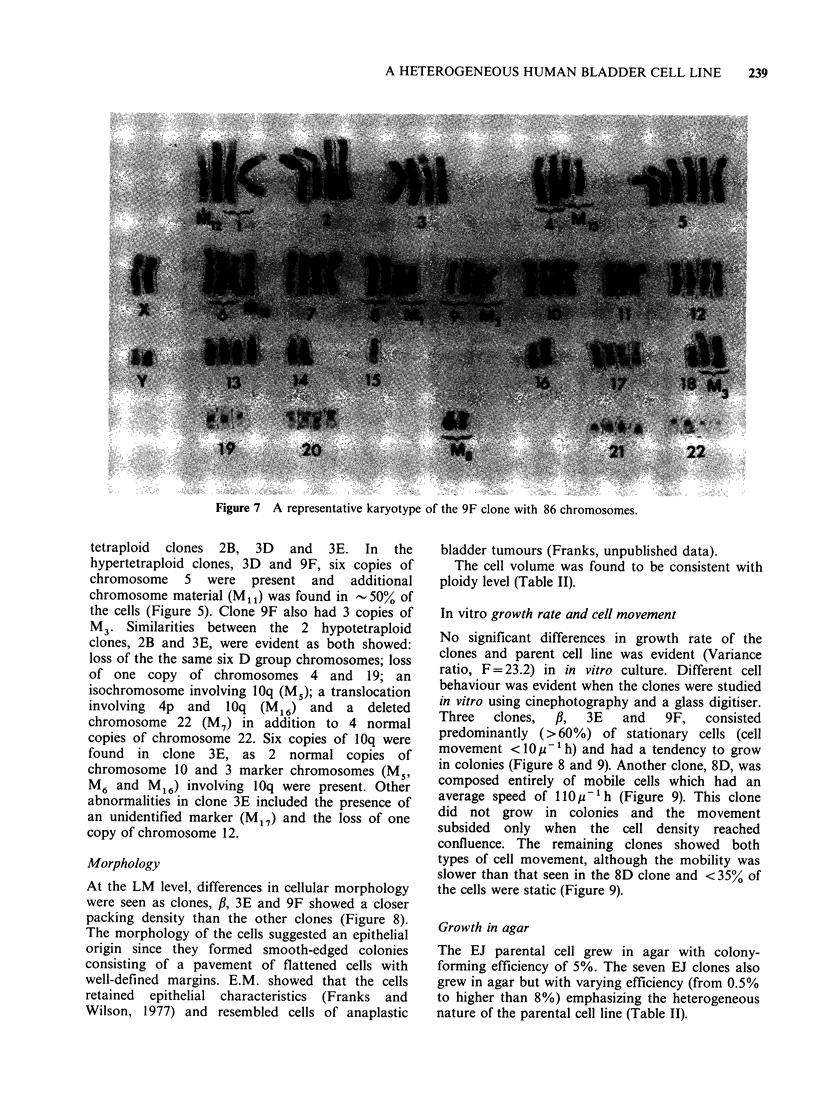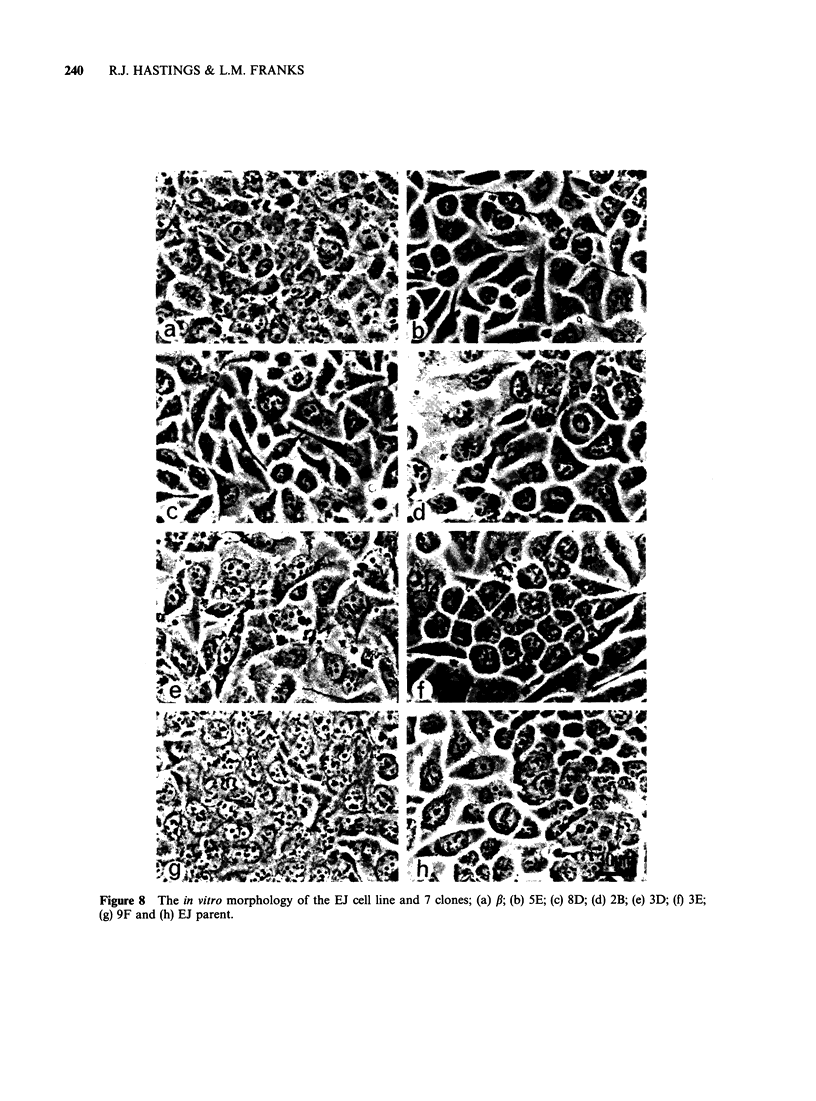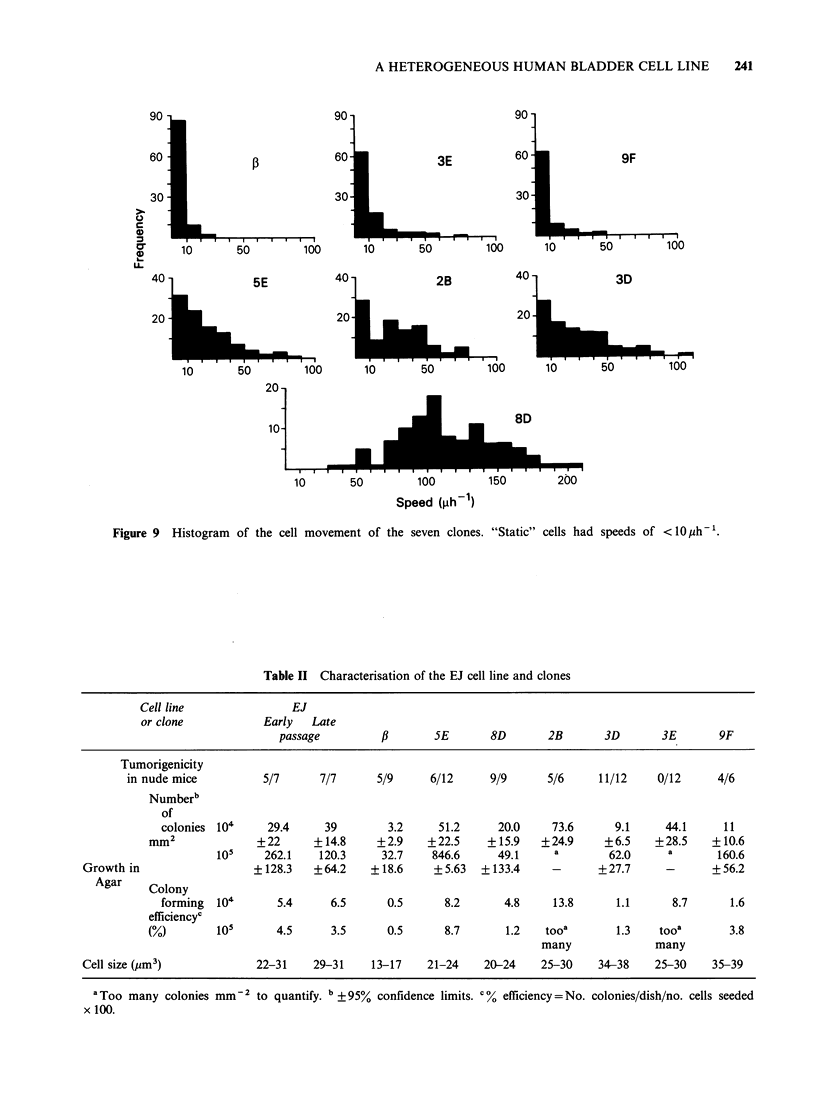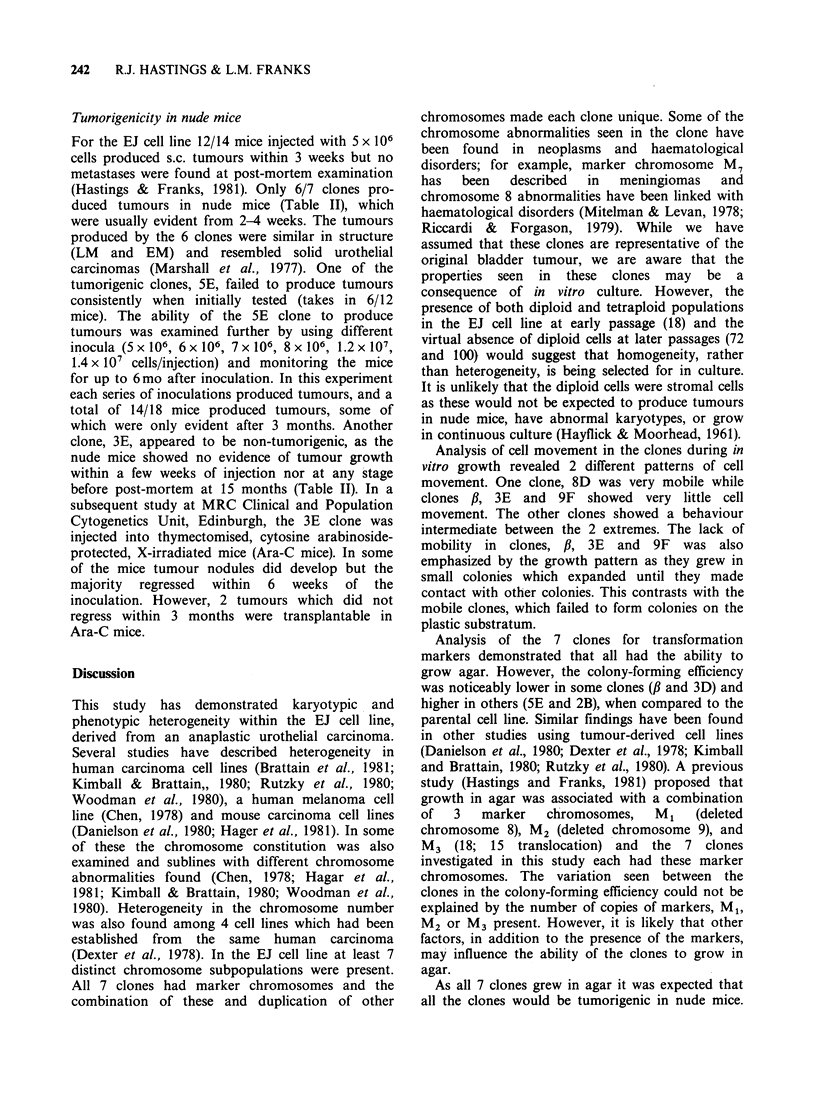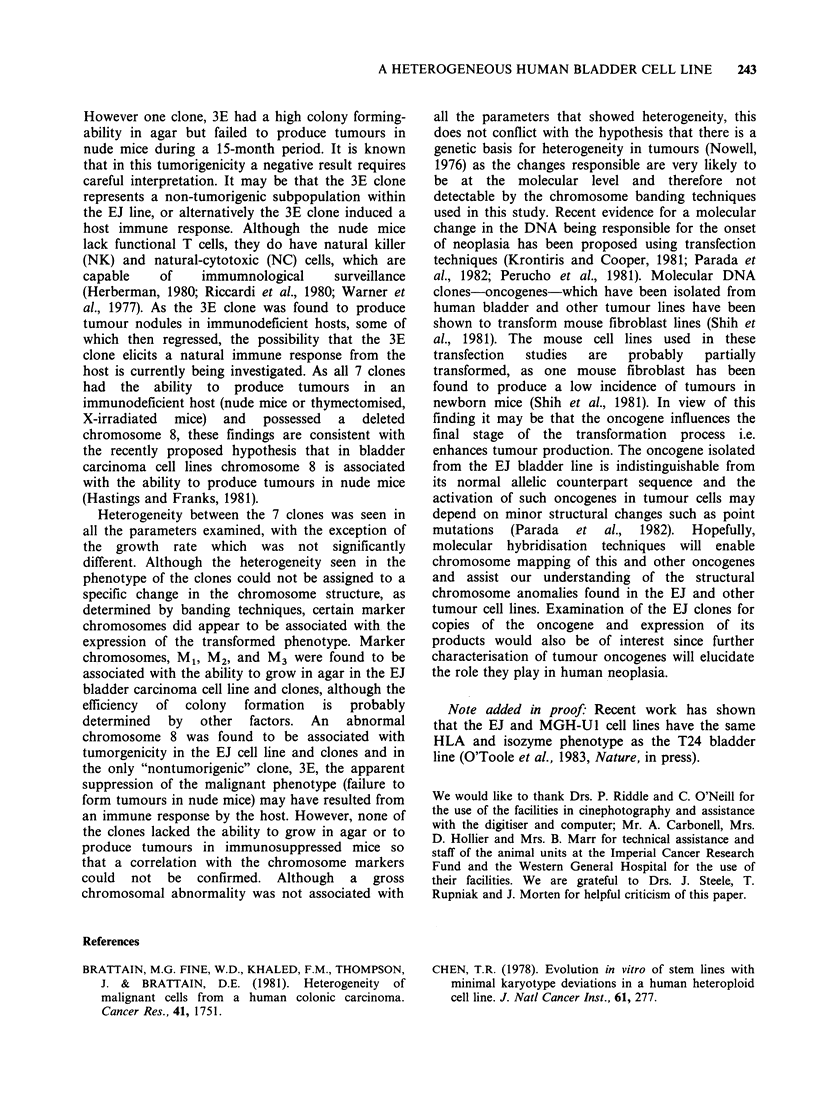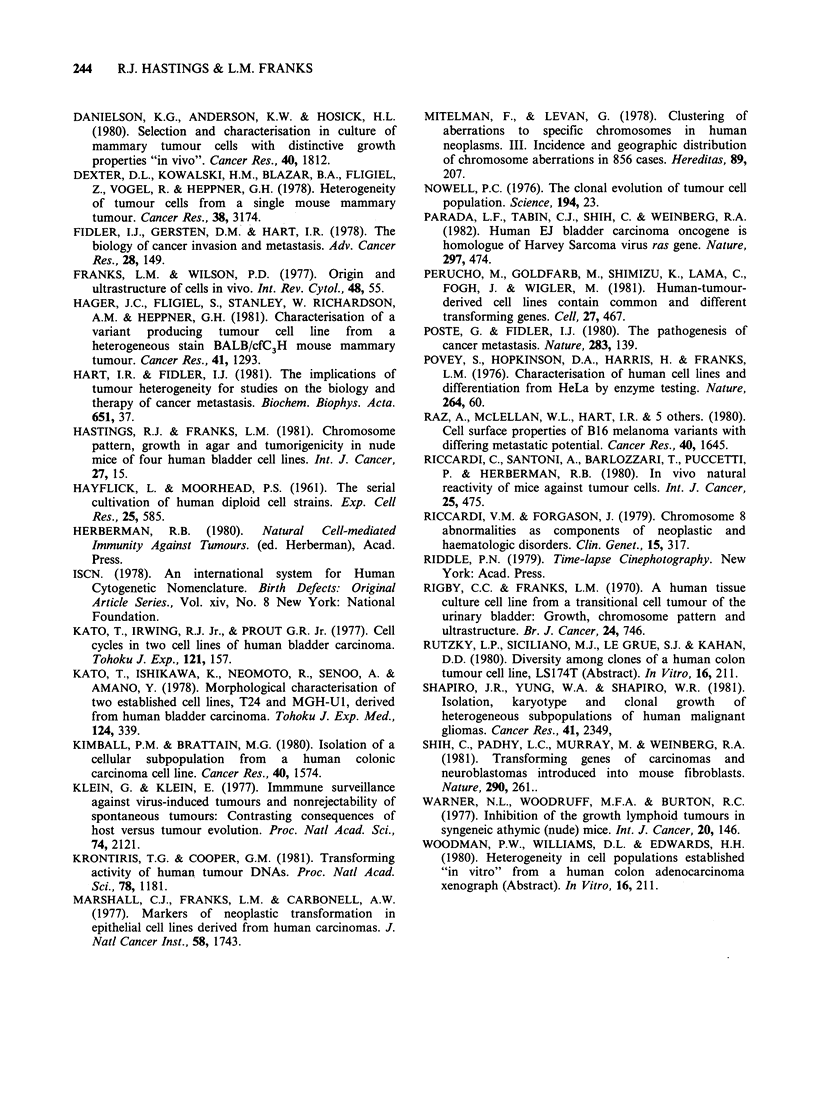# Cellular heterogeneity in a tissue culture cell line derived from a human bladder carcinoma.

**DOI:** 10.1038/bjc.1983.31

**Published:** 1983-02

**Authors:** R. J. Hastings, L. M. Franks

## Abstract

**Images:**


Br. J. Cancer (1983), 47, 233–244

### Cellular heterogeneity in a tissue culture cell line derived from a human bladder carcinoma

R.J. Hastings & L.M. Franks[1]

*Imperial Cancer Research Fund Lincoln's Inn Fields, London WC2A 3PX, & [1]MRC Clinical and Population Cytogenetics Unit, Western General Hospital, Edinburgh EH4 2XU.*

**Summary** To study heterogeneity in a cell line derived from a human bladder carcinoma (EJ), 7 clones were isolated at low passage and examined for differences in culture behaviour, ability to grow in agar and tumorigenicity in nude mice. The parent EJ line had several distinct chromosome populations (both diploid and tetraploid), grew in agar and produced tumours in nude mice. Three of the clones had pseudodiploid modes and 4 had either hypo- or hypertetraploid modes. The 7 clones had 5 marker chromosomes in common but the combination of other marker chromosomes made each clone unique. No significant difference was found between the clones in the *in vitro* growth rate although analysis of *in vitro* culture behaviour showed heterogeneity in the pattern of cell movement on plastic substratum. Three clones were composed of static cells, one clone had very mobile cells; the other clones had rates of movement intermediate between the two. Differences were also found in the packing density of the cloned cells and in the cell size. All 7 clones grew in agar but heterogeneity was seen between the clones as shown by widely varying colony-forming efficiencies (0.5–13%). One clone had a high colony-forming ability in agar but failed to produce tumours in nude mice. The other clones were tumorigenic regardless of colony-forming efficiency in agar. Specific chromosome abnormalities were found to be associated with growth in agar and tumorigenicity but not with the growth pattern or the rate of movement of the cloned cells in culture.

The development of heterogeneity within a tumour, and the selection of progressively more malignant phenotypes may enable a tumour to become more aggressive and autonomous (Nowell, 1976; Klein and Klein, 1977). The tumour cells show multiple phenotypic changes becoming more anaplastic, increasingly independent of growth controls and more metastatic (Poste and Fidler, 1980). Many aspects of heterogeneity have been found in human and animal tumours including DNA content, chromosome number, antigenicity, drug resistance, growth rate, capacity to produce intra- and extra-cellular proteins and cell surface receptors for lectins (Fidler *et al.*, 1978; Hart & Fidler, 1981; Raz *et al.*, 1980; Shapiro *et al.*, 1981). Cellular heterogeneity has also been studied *in vitro* in cell lines derived from tumours, but only a few studies have reported a cellular heterogeneity similar to that seen in tumours *in vivo* (Brattain *et al.*, 1981; Chen, 1978; Dexter *et al.*, 1978).

In a previous study we examined 4 human bladder cell lines derived from urothelial carcinomas and proposed (i) an association between the ability to grow in agar and the presence of marker chromosomes $M_1$ (deleted chromosome 8), $M_2$ (deleted chromosome 9) and $M_3$ (18; 15 translocation) and (ii) an association between tumorigenicity in nude mice and a structurally-altered chromosome 8 (Hastings and Franks, 1981).

Correspondence: L.M. Franks, Imperial Cancer Research Fund, Lincoln Inn Fields, London WC2A 3PX

Received 28 September 1982; accepted 3 November 1982.

One of these urothelial cell lines (EJ) also had several distinct chromosomes subpopulations (Hastings and Franks, 1981). As few studies have demonstrated heterogeneity in both the karyotype and phenotype of a human cell line (Chen, 1978; Kimball and Brattain, 1980; Woodman *et al.*, 1980), we have attempted to determine whether the cell line heterogeneity is associated with the chromosome constitution. We have cloned subpopulations within the EJ cell line for the cellular phenotype, ability to grow in agar and tumorigenicity in immunodeficient mice, to see if these features reflect the heterogeneity seen in the karyotype (Hastings and Franks, 1981).

### Materials and methods

### *Origin of EJ cell line and clones*

The parental EJ cell line was derived from an anaplastic (Grade III) urothelial carcinoma and obtained from Dr. J. Daly (Massachusetts General Hospital, Boston, Massachusetts, USA). This cell line had a heterogeneous chromosome constitution with both diploid and tetraploid subpopulations which became predominantly tetraploid (80% of the cells) after prolonged culture (Hastings and Franks, 1981). The EJ line has been designated MGH-U1 by Kato *et al* (1977), although karyotypic analysis of MGH-U1 showed only the tetraploid population (Kato *et al.*, 1978). Seven clones designated *β*, 5E, 8D, 2B, 3D, 3E and 9F were obtained from the EJ line, at passage nos. 17 and 18, by picking individual cells with an extended

0007-0920/83/020233-12 $02.00

© The Macmillan Press Ltd., 1983

Pasteur pipette and growing each separately in Microwell plates (Flow Laboratories, Irvine, Scotland). Each clone has been maintained in long-term culture, although the results presented in this study were obtained using cells earlier than the 20th passage.

*Cell culture*

All cells were maintained in Dulbecco's modified Eagle's medium (E4) supplemented with 10% calf serum. Cells were passaged with 0.06% trypsin in versene, at 1:10 or 1:20 split ratios when they reached confluence.

Morphological observations were made of the cultures using phase contrast light microscopy (L.M.) and electron microscopy (E.M.). Cells were prepared for E.M. according to the method described by Rigby and Franks (1970). Cell volume was estimated using a Coulter counter (Model ZB1) fitted with a cell volume distribution analyser (Model P64).

*Chromosome preparation and analysis*

Semi-confluent cultures were treated with Vinblastine (0.01 $\mu^{-1}$ml) for 20–30 min at 37°C., harvested, swollen in 0.07 M KCl for 8–20 min, before fixing and washing in Carnoy's fixative (3:1, methanol:acetic acid). Slide preparations were "aged" for a week before banding. To establish the chromosome number, preparations were stained with 4% Giemsa's stain in distilled water for 8–10 min. Fifty metaphase spreads were scored. A modified G-banding technique was used for chromosome analysis. Slides were places in a solution of 0.1% trypsin in Hank's solution (pH 6.8) for between 12 sec and 2 min, and then in Hank's solution (pH 5) for 1 min before staining with 4% Giemsa's stain for 8–10 min. The slide preparations were then washed in distilled water, air-dried and mounted in DePex. Twenty G-banded cells were analysed to ascertain the karyotype.

In vitro *growth rate and cell movement*

The number of cells on a series of duplicate dishes plated with $10^5$ cells was counted daily using a Coulter counter over a period of 6–8 days to measure cell proliferation. The regression coefficients for each clone were compared using Analysis of Variance.

Cell movement was analysed using an Olympus time lapse control (Model PM-ACM). The cells were photographed every 3 min. using a low magnification (×4) lens (Riddle, 1979). The cell movement was quantified by monitoring 100 cells for a period of 4 (film) hours using a LW analytic projector (CLW Ltd., Woodland Hills, Calif., USA), a Hewlett Packard glass digitiser and a Hewlett Packard computer (Hewlett Packard, Palo Alto, Calif., USA).

*Growth in agar*

Cells were seeded in 0.33% Agar in medium on to duplicate 0.5% agar bases at cell densities of $10^5$ and $10^4$ cells per 5 cm dish and colonies >0.1 mm in diameter were scored with an inverted microscope fitted with an image-shearing eyepiece (Watson, M.E.L. Equipment Ltd., Barnet, UK). Details of techniques have been published previously (Marshall *et al.*, 1977). As some variability between colony forming ability was seen between consecutive experiments with the same clone, each assay was repeated ⩾10 times.

*Nude mouse inoculation*

The nude mice (nu-nu) were offspring of heterozygous (nu+) females mated with homozygous nude (nu-nu) males. They were kept under SPF conditions throughout the experiments.

Male nude mice were injected s.c. with $6\times10^6$ cells and where tumours were not evident after 3 months a second series of mice was inoculated. When tumours were >0.5 cm in diameter the mice were killed and tumour samples were fixed for histology and electron microscopy. Paraffin sections were used for L.M., glutaraldehyde-osmic acid-fixed tissues were embedded in Araldite for E.M. (Marshall *et al.*, 1977).

*Isozymes*

The following isozymes were analysed by Dr. S. Povey (Galton Labs., London, UK): $PGM_1$, $PGM_3$: first and third loci of phosphoglucomutase; $GOT_m$: mitochondrial glutamate—oxaloacetate transaminase; G6PD: glucose-6-phosphate dehydrogenase; ESD: esterase D; ADA: adenosine deaminase; ACP: acid phosphatase, "red cell type"; LDH A, LDH B; lactate dehydrogenase, locus A & B. Analysis of the 7 clones showed that the polymorphic isozymes present were the same in phenotype as the EJ parent line (Povey *et al.*, 1976). The cells differed from HeLa in the G6PD phenotype.

**Results**

Seven clones were isolated from the EJ line; one clone, β, was isolated at passage 17, whereas the others, 5E, 8D, 2B, 3D, 3E and 9F were isolated at passage 18.

*Chromosome analysis*

1. *Chromosome number* In the early culture of the EJ cell line (passages 15 and 22), 55% of the cells were near-diploid, with a mode of 46–47 and the remaining 45% were hypotetraploid, with a mode of 90–92. With passage the proportion of hypotetraploid cells increased so that at passage 72 and 100 only 20% of the population was diploid. Cloning of the EJ cell line at early passages selected both diploid and tetraploid clones, all of which had narrow chromosome range and a distinct mode. Clone β was near-diploid with a modal chromosome number of 47 (60%) and a range of 41–49. Clones 5E and 8D were pseudodiploid, with 46 chromosomes seen in 40% and 50% of the cells respectively, and ranges of 40–49 and 41–46 respectively. Two of the tetraploid clones, 2B and 3E, had hypotetraploid modes of 88 (43%) and 90 (30%) and ranges of 76–91 and 80–92 respectively. The other tetraploid clones, 3D and 9F were hypertetraploid having chromosome modes of 96 (22%) and 94 (26%) and ranges of 83–97 and 52–97 respectively. The chromosome range of the cloned cell lines were representative of the early passage parental cell line which had a range of 40–96.

2. *Karyology* Representative karyotypes of the seven clones are shown in Figures 1–7 and detailed in Table I. The chromosome markers which characterised the 7 clones present in the EJ line are considered separately below. The short system for designating structural aberrations has been used except for unbalanced translocations which are described using the detailed system (ISCN, 1978). Numerical changes were also apparent in some of the clones with duplication of some of the normal and abnormal chromosomes occurring in the tetraploid chromosome population (Table 1).

$M_1$ = del (8) (p21)

$M_2$ = del (9) (p13)

$M_3$ = t (15:18) (15qter→15q21::18p11→18qter)

$M_4$ = dup (2) (q31q36)

$M_5$ = i (10q)

$M_6$ = del(10) (p11)

$M_7$ = del(22) (q11)

$M_8$ = unidentified

$M_9$ = i (1p)

$M_{10}$ = i (2q)

$M_{11}$ = unidentified

$M_{12}$ = del (1) (p22p32)

$M_{13}$ = del(4) (q28)

$M_{14}$ = del(X) (q21)

$M_{15}$ = t(6;?) (6qter→6q27::?)

$M_{16}$ = t(4;10) (4pter→cen→10qter)

$M_{17}$ = unidentified

$M_{18}$ = t(6;14) (6pter→cen→14 qter)

Some of the marker chromosomes were found ⩾50% of the cells of the EJ line ($M_1$, $M_2$, $M_3$, $M_5$, $M_7$, $M_8$, $M_{13}$) whereas markers $M_9$, $M_{10}$, $M_{14}$, $M_{15}$ and $M_{17}$) were only seen in 10% of the cells examined in the EJ line. Some of the marker chromosomes seen in the clones were not initially seen in the parental cell line but further examination confirmed the presence of the marker in the EJ line and showed that the marker chromosomes characteristic for each clone were selected rather than induced (Table I). Five marker chromosomes, $M_1$, $M_2$, $M_3$, $M_8$ and $M_{13}$ were present in all 7 clones, and additional copies of either chromosome 19 and/or 20 were found in the

**Table I** Karyotypes of the EJ Clones

| *Clone* | *Karyotype* |
|---|---|
| β | 47, XY, −4, +5, −8, −9, −10, −15, −18, +$M_1$ +$M_2$, +$M_3$, +$M_5$, +$M_8$, +$M_{13}$. |
| 5E | 46, XY, −2, −4, −8, −9, −10, −15, −18, −X, +$M_1$, +$M_2$, +$M_3$, +$M_4$, +$M_5$, +$M_8$, +$M_{13}$, $M_{14}$. |
| 8D | 46, XY, −4, −8, −9, −10, −15, −18, −22, +$M_1$, +$M_2$, +$M_3$, +$M_6$, +$M_7$, +$M_8$, +$M_{13}$. |
| 2B | 88, XXYY, −4, −4, −8, −8, −9, −9, −10, −13, −13, −14, −15, −15, −15, −18, −18, −19, +20, +$M_1$, +$M_1$, +$M_2$, +$M_2$, +$M_3$, +$M_3$, +$M_7$, +$M_8$, +$M_8$, +$M_{13}$, +$M_{16}$. |
| 3D | 96, XXYY, −4, −4, +5, +5, −6, −8, −8, −9, −9, −15, −15, −18, −18, +19, +20, +$M_1$, +$M_1$, +$M_2$, +$M_2$, +$M_3$, +$M_3$, +$M_8$, +$M_8$, +$M_{13}$, +$M_{13}$, +$M_{15}$. |
| 3E | 90, XXYY, −2, −4, −4, −8, −8, −9, −9, −10, −10, −12, −13, −13, −14, −15, −15, −15, −18, −18, −19, +20, +20, +$M_1$, +$M_1$, +$M_2$, +$M_2$, +$M_3$, +$M_3$, +$M_5$, +$M_6$, +$M_7$, +$M_8$, +$M_8$, +$M_{10}$, +$M_{13}$, +$M_{16}$. |
| 9F | 94, XXYY, −1, −4, −4, +5, +5, −6, −8, −9, −9, −14, −15, −15, −18, −18, +$M_1$, +$M_1$, +$M_2$, +$M_2$, +$M_3$, +$M_3$, +$M_3$, +$M_8$, +$M_8$, +$M_{12}$, +$M_{13}$, +$M_{13}$, +$M_{18}$. |

**Figure 1** A representative karyotype of the $\beta$ clone with 46 chromosomes. Marker, $M_8$, was missing from this particular spread.

**Figure 2** A representative karyotype of the 5E clone with 46 chromosomes.

**Figure 3** A representative karyotype of the 8D clone with 46 chromosomes.

**Figure 4** A representative karyotype of the 2B clone with 87 chromosomes.

**Figure 5** A representative karyotype of the 3D clone with 93 chromosomes.

**Figure 6** A representative karyotype of the 3E clone with 84 chromosomes.

**Figure 7** A representative karyotype of the 9F clone with 86 chromosomes.

tetraploid clones 2B, 3D and 3E. In the hypertetraploid clones, 3D and 9F, six copies of chromosome 5 were present and additional chromosome material ($M_{11}$) was found in $\sim 50\%$ of the cells (Figure 5). Clone 9F also had 3 copies of $M_3$. Similarities between the 2 hypotetraploid clones, 2B and 3E, were evident as both showed: loss of the the same six D group chromosomes; loss of one copy of chromosomes 4 and 19; an isochromosome involving 10q ($M_5$); a translocation involving 4p and 10q ($M_{16}$) and a deleted chromosome 22 ($M_7$) in addition to 4 normal copies of chromosome 22. Six copies of 10q were found in clone 3E, as 2 normal copies of chromosome 10 and 3 marker chromosomes ($M_5$, $M_6$ and $M_{16}$) involving 10q were present. Other abnormalities in clone 3E included the presence of an unidentified marker ($M_{17}$) and the loss of one copy of chromosome 12.

### *Morphology*

At the LM level, differences in cellular morphology were seen as clones, *β*, 3E and 9F showed a closer packing density than the other clones (Figure 8). The morphology of the cells suggested an epithelial origin since they formed smooth-edged colonies consisting of a pavement of flattened cells with well-defined margins. E.M. showed that the cells retained epithelial characteristics (Franks and Wilson, 1977) and resembled cells of anaplastic bladder tumours (Franks, unpublished data).

The cell volume was found to be consistent with ploidy level (Table II).

### In vitro *growth rate and cell movement*

No significant differences in growth rate of the clones and parent cell line was evident (Variance ratio, $F = 23.2$) in *in vitro* culture. Different cell behaviour was evident when the clones were studied *in vitro* using cinephotography and a glass digitiser. Three clones, *β*, 3E and 9F, consisted predominantly (>60%) of stationary cells (cell movement $< 10\,\mu^{-1}$ h) and had a tendency to grow in colonies (Figure 8 and 9). Another clone, 8D, was composed entirely of mobile cells which had an average speed of $110\,\mu^{-1}$ h (Figure 9). This clone did not grow in colonies and the movement subsided only when the cell density reached confluence. The remaining clones showed both types of cell movement, although the mobility was slower than that seen in the 8D clone and <35% of the cells were static (Figure 9).

### *Growth in agar*

The EJ parental cell grew in agar with colony-forming efficiency of 5%. The seven EJ clones also grew in agar but with varying efficiency (from 0.5% to higher than 8%) emphasizing the heterogeneous nature of the parental cell line (Table II).

**Figure 8** The *in vitro* morphology of the EJ cell line and 7 clones; (a) *β*; (b) 5E; (c) 8D; (d) 2B; (e) 3D; (f) 3E; (g) 9F and (h) EJ parent.

**Figure 9** Histogram of the cell movement of the seven clones. "Static" cells had speeds of $<10\mu h^{-1}$.

**Table II** Characterisation of the EJ cell line and clones

| *Cell line or clone* | | *EJ Early passage* | *EJ Late passage* | *β* | *5E* | *8D* | *2B* | *3D* | *3E* | *9F* |
|---|---|---|---|---|---|---|---|---|---|---|
| Tumorigenicity in nude mice | | 5/7 | 7/7 | 5/9 | 6/12 | 9/9 | 5/6 | 11/12 | 0/12 | 4/6 |
| Growth in Agar: Number[b] of colonies mm$^2$ | $10^4$ | 29.4 ±22 | 39 ±14.8 | 3.2 ±2.9 | 51.2 ±22.5 | 20.0 ±15.9 | 73.6 ±24.9 | 9.1 ±6.5 | 44.1 ±28.5 | 11 ±10.6 |
| | $10^5$ | 262.1 ±128.3 | 120.3 ±64.2 | 32.7 ±18.6 | 846.6 ±5.63 | 49.1 ±133.4 | [a] – | 62.0 ±27.7 | [a] – | 160.6 ±56.2 |
| Growth in Agar: Colony forming efficiency[c] (%) | $10^4$ | 5.4 | 6.5 | 0.5 | 8.2 | 4.8 | 13.8 | 1.1 | 8.7 | 1.6 |
| | $10^5$ | 4.5 | 3.5 | 0.5 | 8.7 | 1.2 | too[a] many | 1.3 | too[a] many | 3.8 |
| Cell size ($\mu m^3$) | | 22–31 | 29–31 | 13–17 | 21–24 | 20–24 | 25–30 | 34–38 | 25–30 | 35–39 |

[a]Too many colonies mm$^{-2}$ to quantify. [b]±95% confidence limits. [c]% efficiency = No. colonies/dish/no. cells seeded ×100.

*Tumorigenicity in nude mice*

For the EJ cell line 12/14 mice injected with $5 \times 10^6$ cells produced s.c. tumours within 3 weeks but no metastases were found at post-mortem examination (Hastings & Franks, 1981). Only 6/7 clones produced tumours in nude mice (Table II), which were usually evident from 2–4 weeks. The tumours produced by the 6 clones were similar in structure (LM and EM) and resembled solid urothelial carcinomas (Marshall *et al.,* 1977). One of the tumorigenic clones, 5E, failed to produce tumours consistently when initially tested (takes in 6/12 mice). The ability of the 5E clone to produce tumours was examined further by using different inocula ($5 \times 10^6$, $6 \times 10^6$, $7 \times 10^6$, $8 \times 10^6$, $1.2 \times 10^7$, $1.4 \times 10^7$ cells/injection) and monitoring the mice for up to 6 mo after inoculation. In this experiment each series of inoculations produced tumours, and a total of 14/18 mice produced tumours, some of which were only evident after 3 months. Another clone, 3E, appeared to be non-tumorigenic, as the nude mice showed no evidence of tumour growth within a few weeks of injection nor at any stage before post-mortem at 15 months (Table II). In a subsequent study at MRC Clinical and Population Cytogenetics Unit, Edinburgh, the 3E clone was injected into thymectomised, cytosine arabinoside-protected, X-irradiated mice (Ara-C mice). In some of the mice tumour nodules did develop but the majority regressed within 6 weeks of the inoculation. However, 2 tumours which did not regress within 3 months were transplantable in Ara-C mice.

### Discussion

This study has demonstrated karyotypic and phenotypic heterogeneity within the EJ cell line, derived from an anaplastic urothelial carcinoma. Several studies have described heterogeneity in human carcinoma cell lines (Brattain *et al.,* 1981; Kimball & Brattain,, 1980; Rutzky *et al.,* 1980; Woodman *et al.,* 1980), a human melanoma cell line (Chen, 1978) and mouse carcinoma cell lines (Danielson *et al.*, 1980; Hager *et al.*, 1981). In some of these the chromosome constitution was also examined and sublines with different chromosome abnormalities found (Chen, 1978; Hagar *et al.,* 1981; Kimball & Brattain, 1980; Woodman *et al.,* 1980). Heterogeneity in the chromosome number was also found among 4 cell lines which had been established from the same human carcinoma (Dexter *et al.,* 1978). In the EJ cell line at least 7 distinct chromosome subpopulations were present. All 7 clones had marker chromosomes and the combination of these and duplication of other chromosomes made each clone unique. Some of the chromosome abnormalities seen in the clone have been found in neoplasms and haematological disorders; for example, marker chromosome $M_7$ has been described in meningiomas and chromosome 8 abnormalities have been linked with haematological disorders (Mitelman & Levan, 1978; Riccardi & Forgason, 1979). While we have assumed that these clones are representative of the original bladder tumour, we are aware that the properties seen in these clones may be a consequence of *in vitro* culture. However, the presence of both diploid and tetraploid populations in the EJ cell line at early passage (18) and the virtual absence of diploid cells at later passages (72 and 100) would suggest that homogeneity, rather than heterogeneity, is being selected for in culture. It is unlikely that the diploid cells were stromal cells as these would not be expected to produce tumours in nude mice, have abnormal karyotypes, or grow in continuous culture (Hayflick & Moorhead, 1961).

Analysis of cell movement in the clones during *in vitro* growth revealed 2 different patterns of cell movement. One clone, 8D was very mobile while clones $\beta$, 3E and 9F showed very little cell movement. The other clones showed a behaviour intermediate between the 2 extremes. The lack of mobility in clones, $\beta$, 3E and 9F was also emphasized by the growth pattern as they grew in small colonies which expanded until they made contact with other colonies. This contrasts with the mobile clones, which failed to form colonies on the plastic substratum.

Analysis of the 7 clones for transformation markers demonstrated that all had the ability to grow agar. However, the colony-forming efficiency was noticeably lower in some clones ($\beta$ and 3D) and higher in others (5E and 2B), when compared to the parental cell line. Similar findings have been found in other studies using tumour-derived cell lines (Danielson *et al.*, 1980; Dexter *et al.*, 1978; Kimball and Brattain, 1980; Rutzky *et al.*, 1980). A previous study (Hastings and Franks, 1981) proposed that growth in agar was associated with a combination of 3 marker chromosomes, $M_1$ (deleted chromosome 8), $M_2$ (deleted chromosome 9), and $M_3$ (18; 15 translocation) and the 7 clones investigated in this study each had these marker chromosomes. The variation seen between the clones in the colony-forming efficiency could not be explained by the number of copies of markers, $M_1$, $M_2$ or $M_3$ present. However, it is likely that other factors, in addition to the presence of the markers, may influence the ability of the clones to grow in agar.

As all 7 clones grew in agar it was expected that all the clones would be tumorigenic in nude mice.

However one clone, 3E had a high colony forming-ability in agar but failed to produce tumours in nude mice during a 15-month period. It is known that in this tumorigenicity a negative result requires careful interpretation. It may be that the 3E clone represents a non-tumorigenic subpopulation within the EJ line, or alternatively the 3E clone induced a host immune response. Although the nude mice lack functional T cells, they do have natural killer (NK) and natural-cytotoxic (NC) cells, which are capable of immumnological surveillance (Herberman, 1980; Riccardi *et al.*, 1980; Warner *et al.*, 1977). As the 3E clone was found to produce tumour nodules in immunodeficient hosts, some of which then regressed, the possibility that the 3E clone elicits a natural immune response from the host is currently being investigated. As all 7 clones had the ability to produce tumours in an immunodeficient host (nude mice or thymectomised, X-irradiated mice) and possessed a deleted chromosome 8, these findings are consistent with the recently proposed hypothesis that in bladder carcinoma cell lines chromosome 8 is associated with the ability to produce tumours in nude mice (Hastings and Franks, 1981).

Heterogeneity between the 7 clones was seen in all the parameters examined, with the exception of the growth rate which was not significantly different. Although the heterogeneity seen in the phenotype of the clones could not be assigned to a specific change in the chromosome structure, as determined by banding techniques, certain marker chromosomes did appear to be associated with the expression of the transformed phenotype. Marker chromosomes, $M_1$, $M_2$, and $M_3$ were found to be associated with the ability to grow in agar in the EJ bladder carcinoma cell line and clones, although the efficiency of colony formation is probably determined by other factors. An abnormal chromosome 8 was found to be associated with tumorgenicity in the EJ cell line and clones and in the only "nontumorigenic" clone, 3E, the apparent suppression of the malignant phenotype (failure to form tumours in nude mice) may have resulted from an immune response by the host. However, none of the clones lacked the ability to grow in agar or to produce tumours in immunosuppressed mice so that a correlation with the chromosome markers could not be confirmed. Although a gross chromosomal abnormality was not associated with all the parameters that showed heterogeneity, this does not conflict with the hypothesis that there is a genetic basis for heterogeneity in tumours (Nowell, 1976) as the changes responsible are very likely to be at the molecular level and therefore not detectable by the chromosome banding techniques used in this study. Recent evidence for a molecular change in the DNA being responsible for the onset of neoplasia has been proposed using transfection techniques (Krontiris and Cooper, 1981; Parada *et al.*, 1982; Perucho *et al.*, 1981). Molecular DNA clones—oncogenes—which have been isolated from human bladder and other tumour lines have been shown to transform mouse fibroblast lines (Shih *et al.*, 1981). The mouse cell lines used in these transfection studies are probably partially transformed, as one mouse fibroblast has been found to produce a low incidence of tumours in newborn mice (Shih *et al.*, 1981). In view of this finding it may be that the oncogene influences the final stage of the transformation process i.e. enhances tumour production. The oncogene isolated from the EJ bladder line is indistinguishable from its normal allelic counterpart sequence and the activation of such oncogenes in tumour cells may depend on minor structural changes such as point mutations (Parada *et al.*, 1982). Hopefully, molecular hybridisation techniques will enable chromosome mapping of this and other oncogenes and assist our understanding of the structural chromosome anomalies found in the EJ and other tumour cell lines. Examination of the EJ clones for copies of the oncogene and expression of its products would also be of interest since further characterisation of tumour oncogenes will elucidate the role they play in human neoplasia.

*Note added in proof:* Recent work has shown that the EJ and MGH-U1 cell lines have the same HLA and isozyme phenotype as the T24 bladder line (O'Toole *et al.*, 1983, *Nature*, in press).

We would like to thank Drs. P. Riddle and C. O'Neill for the use of the facilities in cinephotography and assistance with the digitiser and computer; Mr. A. Carbonell, Mrs. D. Hollier and Mrs. B. Marr for technical assistance and staff of the animal units at the Imperial Cancer Research Fund and the Western General Hospital for the use of their facilities. We are grateful to Drs. J. Steele, T. Rupniak and J. Morten for helpful criticism of this paper.

**References**

BRATTAIN, M.G. FINE, W.D., KHALED, F.M., THOMPSON, J. & BRATTAIN, D.E. (1981). Heterogeneity of malignant cells from a human colonic carcinoma. *Cancer Res.*, **41**, 1751.

CHEN, T.R. (1978). Evolution *in vitro* of stem lines with minimal karyotype deviations in a human heteroploid cell line. *J. Natl Cancer Inst.*, **61**, 277.

DANIELSON, K.G., ANDERSON, K.W. & HOSICK, H.L. (1980). Selection and characterisation in culture of mammary tumour cells with distinctive growth properties "in vivo". *Cancer Res.*, **40,** 1812.

DEXTER, D.L., KOWALSKI, H.M., BLAZAR, B.A., FLIGIEL, Z., VOGEL, R. & HEPPNER, G.H. (1978). Heterogeneity of tumour cells from a single mouse mammary tumour. *Cancer Res.*, **38,** 3174.

FIDLER, I.J., GERSTEN, D.M. & HART, I.R. (1978). The biology of cancer invasion and metastasis. *Adv. Cancer Res.*, **28,** 149.

FRANKS, L.M. & WILSON, P.D. (1977). Origin and ultrastructure of cells in vivo. *Int. Rev. Cytol.*, **48,** 55.

HAGER, J.C., FLIGIEL, S., STANLEY, W. RICHARDSON, A.M. & HEPPNER, G.H. (1981). Characterisation of a variant producing tumour cell line from a heterogeneous stain BALB/cf$C_3$H mouse mammary tumour. *Cancer Res.*, **41,** 1293.

HART, I.R. & FIDLER, I.J. (1981). The implications of tumour heterogeneity for studies on the biology and therapy of cancer metastasis. *Biochem. Biophys. Acta.* **651,** 37.

HASTINGS, R.J. & FRANKS, L.M. (1981). Chromosome pattern, growth in agar and tumorigenicity in nude mice of four human bladder cell lines. *Int. J. Cancer,* **27,** 15.

HAYFLICK, L. & MOORHEAD, P.S. (1961). The serial cultivation of human diploid cell strains. *Exp. Cell Res.*, **25,** 585.

HERBERMAN, R.B. (1980). *Natural Cell-mediated Immunity Against Tumours.* (ed. Herberman), Acad. Press.

ISCN. (1978). An international system for Human Cytogenetic Nomenclature. *Birth Defects: Original Article Series.*, Vol. xiv, No. 8 New York: National Foundation.

KATO, T., IRWING, R.J. Jr., & PROUT G.R. Jr. (1977). Cell cycles in two cell lines of human bladder carcinoma. *Tohoku J. Exp.*, **121,** 157.

KATO, T., ISHIKAWA, K., NEOMOTO, R., SENOO, A. & AMANO, Y. (1978). Morphological characterisation of two established cell lines, T24 and MGH-U1, derived from human bladder carcinoma. *Tohoku J. Exp. Med.*, **124,** 339.

KIMBALL, P.M. & BRATTAIN, M.G. (1980). Isolation of a cellular subpopulation from a human colonic carcinoma cell line. *Cancer Res.*, **40,** 1574.

KLEIN, G. & KLEIN, E. (1977). Immmune surveillance against virus-induced tumours and nonrejectability of spontaneous tumours: Contrasting consequences of host versus tumour evolution. *Proc. Natl Acad. Sci.*, **74,** 2121.

KRONTIRIS, T.G. & COOPER, G.M. (1981). Transforming activity of human tumour DNAs. *Proc. Natl Acad. Sci.*, **78,** 1181.

MARSHALL, C.J., FRANKS, L.M. & CARBONELL, A.W. (1977). Markers of neoplastic transformation in epithelial cell lines derived from human carcinomas. *J. Natl Cancer Inst.*, **58,** 1743.

MITELMAN, F., & LEVAN, G. (1978). Clustering of aberrations to specific chromosomes in human neoplasms. III. Incidence and geographic distribution of chromosome aberrations in 856 cases. *Hereditas,* **89,** 207.

NOWELL, P.C. (1976). The clonal evolution of tumour cell population. *Science,* **194,** 23.

PARADA, L.F., TABIN, C.J., SHIH, C. & WEINBERG, R.A. (1982). Human EJ bladder carcinoma oncogene is homologue of Harvey Sarcoma virus *ras* gene. *Nature,* **297,** 474.

PERUCHO, M., GOLDFARB, M., SHIMIZU, K., LAMA, C., FOGH, J. & WIGLER, M. (1981). Human-tumour-derived cell lines contain common and different transforming genes. *Cell,* **27,** 467.

POSTE, G. & FIDLER, I.J. (1980). The pathogenesis of cancer metastasis. *Nature,* **283,** 139.

POVEY, S., HOPKINSON, D.A., HARRIS, H. & FRANKS, L.M. (1976). Characterisation of human cell lines and differentiation from HeLa by enzyme testing. *Nature,* **264,** 60.

RAZ, A., McLELLAN, W.L., HART, I.R. & 5 others. (1980). Cell surface properties of B16 melanoma variants with differing metastatic potential. *Cancer Res.*, **40,** 1645.

RICCARDI, C., SANTONI, A., BARLOZZARI, T., PUCCETTI, P. & HERBERMAN, R.B. (1980). In vivo natural reactivity of mice against tumour cells. *Int. J. Cancer,* **25,** 475.

RICCARDI, V.M. & FORGASON, J. (1979). Chromosome 8 abnormalities as components of neoplastic and haematologic disorders. *Clin. Genet.*, **15,** 317.

RIDDLE, P.N. (1979). *Time-lapse Cinephotography.* New York: Acad. Press.

RIGBY, C.C. & FRANKS, L.M. (1970). A human tissue culture cell line from a transitional cell tumour of the urinary bladder: Growth, chromosome pattern and ultrastructure. *Br. J. Cancer,* **24,** 746.

RUTZKY, L.P., SICILIANO, M.J., LE GRUE, S.J. & KAHAN, D.D. (1980). Diversity among clones of a human colon tumour cell line, LS174T (Abstract). *In Vitro,* **16,** 211.

SHAPIRO, J.R., YUNG, W.A. & SHAPIRO, W.R. (1981). Isolation, karyotype and clonal growth of heterogeneous subpopulations of human malignant gliomas. *Cancer Res.*, **41,** 2349,

SHIH, C., PADHY, L.C., MURRAY, M. & WEINBERG, R.A. (1981). Transforming genes of carcinomas and neuroblastomas introduced into mouse fibroblasts. *Nature,* **290,** 261..

WARNER, N.L., WOODRUFF, M.F.A. & BURTON, R.C. (1977). Inhibition of the growth lymphoid tumours in syngeneic athymic (nude) mice. *Int. J. Cancer,* **20,** 146.

WOODMAN, P.W., WILLIAMS, D.L. & EDWARDS, H.H. (1980). Heterogeneity in cell populations established "in vitro" from a human colon adenocarcinoma xenograph (Abstract). *In Vitro,* **16,** 211.